# Performance of BioFire Blood Culture Identification 2 Panel (BCID2) for the detection of bloodstream pathogens and their associated resistance markers: a systematic review and meta-analysis of diagnostic test accuracy studies

**DOI:** 10.1186/s12879-022-07772-x

**Published:** 2022-10-20

**Authors:** Anna Maria Peri, Weiping Ling, Luis Furuya-Kanamori, Patrick N. A. Harris, David L. Paterson

**Affiliations:** 1 University of Queensland Centre for Clinical Research, Herston, QLD 4029 Australia; 2 Central Microbiology, Pathology Queensland, Royal Brisbane and Women’s Hospital, Herston, QLD 4029 Australia; 3 Infectious Diseases Unit, Royal Brisbane and Women’s Hospital, Herston, QLD 4029 Australia

**Keywords:** Bloodstream infection, Sepsis, Blood culture, Molecular diagnostic techniques, BCID2, Meta-analysis

## Abstract

**Background:**

Early identification of bloodstream pathogens and their associated antimicrobial resistance may shorten time to optimal therapy in patients with sepsis. The BioFire Blood Culture Identification 2 Panel (BCID2) is a novel multiplex PCR detecting 43 targets directly from positive blood cultures, reducing turnaround times.

**Methods:**

We have performed a systematic review and meta-analysis of diagnostic test accuracy studies to assess the BCID2 performance for pathogen identification and resistance markers detection compared to gold standard culture-based methods (including phenotypic and/or genotypic characterization).

**Results:**

Nine studies were identified reporting data to build 2 × 2 tables for each BCID2 target, including 2005 blood cultures. The pooled specificity of the assay was excellent (> 97%) across most subgroups of targets investigated, with a slightly broader confidence interval for *S. epidermidis* (98.1%, 95% CI 93.1 to 99.5). Pooled sensitivity was also high for the major determinants of bloodstream infection, including *Enterobacterales* (98.2%, 95% CI 96.3 to 99.1)*, S. aureus* (96.0%, 95% CI 90.4 to 98.4)*, Streptococcus spp*. (96.7%, 95% CI 92.8 to 98.5), *P. aeruginosa* (92.7%, 95% CI 83.1 to 97.0), *E. faecalis* (92.3%, 95% CI 83.5 to 96.6), as well as *bla*_CTX-M_ (94.9, 95% CI 85.7 to 98.3), carbapenemases (94.9%, 95% CI 83.4 to 98.6) and *mecA/C & MREJ* (93.9%, 95% CI 83.0 to 98.0). Sensitivity for less common targets was slightly lower, possibly due to their under-representation in the included studies.

**Conclusions:**

BCID2 showed good performance for detecting major determinants of bloodstream infection and could support early antimicrobial treatment, especially for ESBL or carbapenemase-producing Gram-negative bacilli and methicillin-resistant *S. aureus*.

**Supplementary Information:**

The online version contains supplementary material available at 10.1186/s12879-022-07772-x.

## Background

Bloodstream infections and sepsis are a leading cause of death, and early antimicrobial treatment is associated to improved survival [1]. However, the long turnaround times of conventional blood culture methods as well as the increasing burden of antimicrobial resistance often hamper a prompt management of affected patients [2].

The implementation of matrix-assisted laser desorption/ionization time of flight mass spectrometry (MALDI-TOF MS) in clinical laboratories in recent years has substantially contributed to reducing the time to identify bloodstream pathogens. However, up to 12–24 h from blood culture positivity are still required for pathogen characterization, and even longer times are needed before antimicrobial susceptibility testing (AST) is available according to standard methods [3].

Therefore, other molecular technologies are emerging, based on the detection of pathogen DNA directly from positive blood cultures, aimed at further reducing time to results, and simplifying the laboratory workflow [4]. Limitations of such tests still apply, including the ability to detect a limited panel of pathogens and resistance genes only, making their clinical implementation challenging. Among these tests, the BioFire Blood Culture Identification Panel (BCID, bioMérieux) is a multiplex PCR applied on positive blood cultures whose original version was based on the identification of 24 microorganisms and 3 antimicrobial resistance genes (*mecA*, *vanA/B* and *KPC*), and whose use has been associated to early appropriate treatment [5, 6]. Some relevant targets were missing from the original panel of the assay, as highlighted by a study where a subset of bacteraemia cases caused by organisms not detected by the first version of the test were associated to adverse clinical outcomes and mainly caused by anaerobes [7].

To overcome this limitation, a new version of the BCID has been recently released (BioFire Blood Culture Identification 2 Panel, BCID2, bioMerieux) including 33 pathogens and 10 resistance markers, reaching a broad coverage of the most common determinant of bloodstream infection (and the broadest coverage among molecular tests applied on positive blood cultures), and making the implementation of the test in clinical practice more promising [8].

A few studies have been published so far assessing the performance of this test in real-life scenarios, showing relatively good agreement with conventional culture-based testing [9–14]. However, the limited sample size of these individual studies prevented a reliable assessment of the performance of BCID2, and results were often described as percentage of agreement or concordance with conventional testing rather than reporting diagnostic accuracy measures (e.g., sensitivity [Se], specificity [Sp]) that are used in clinical practice [9, 10, 13]. Considering the potential implementation of the BCID2 in clinical practice as a tool for guiding clinical decision making in patients with bloodstream infection in the close future, gaining a better understanding of its diagnostic accuracy in real-life settings could represent a valuable knowledge. Therefore, we have performed a systematic review and meta-analysis of diagnostic test accuracy studies to estimate the performance of the BCID2 for pathogen identification and resistance markers detection compared to conventional blood culture-based methods, including phenotypic and genotypic characterization on culture isolates.

## Methods

These systematic review and meta-analysis are reported according to the Preferred Reporting Items for Systematic Reviews and Meta-Analyses of Diagnostic Test Accuracy Studies (PRISMA-DTA) guideline [15] (Additional file 1: Data 1).

### Search strategy

The search strategy was built by an experienced librarian in Medline, Scopus, Embase as well as in pre-print databases (i.e., MedRxiv, BioRrxiv and SSRN Electronic Journal) on February 4th, 2022, for studies that assessed the performance of the BCID2. The search terms included “BCID2”, “BIOFIRE Blood Culture Identification 2”, “Biofire Filmarray”, “Panel 2”, “BioFire FilmArray Blood Culture Identification 2”, “Blood culture Identification panel 2”.

After identifying eligible papers, a backward & forward citation search was performed in Scopus identifying further papers which also underwent blind screening by the two reviewers. Moreover, on April 12th, 2022, a search of the grey literature was performed using the terms “Biofire Filmarray” and “BCID2”; again, blind screening by the two reviewers was performed. Websites identified through the grey literature search were further searched for additional information.

No restriction in terms of publication date or language were introduced in the systematic search.

The search strategy details are available in the Additional file 1: Data 2.

### Inclusion criteria

To be included the studies had to assess the performance of the BCID2 on prospectively or randomly collected clinical blood cultures samples flagging positive, or spiked blood cultures. Specifically, they had to report information to build a 2 × 2 diagnostic contingency table for each BCID2 target, with blood culture-based conventional methods as the gold standard comparator.

The BCID2 is manufactured by bioMérieux (France) and received Conformité Européenne In vitro Diagnostics (CE IVD), Food and Drug Administration (FDA) and Therapeutic Goods Administration (TGA) approvals in Europe, the U.S and Australia respectively, during 2020. Both studies including the Research Use Only (RUO) versions (or Investigational Use Only, IUO) and the IVD commercial versions of the assay were eligible for inclusion in the meta-analysis since the RUO versions did not differ from the currently commercially approved ones [12]. Differently, studies based on the use of “RUO prototype” versions of the assay were not deemed eligible for inclusion since, as confirmed by the manufacturer, changes were made to the prototypes before developing the definitive RUO/IUO version which is identical to IVD cleared and commercially available one. Targets included in the BCID2 panel are listed in Table 1.

**Table 1:** Targets included in the BCID2 Panel

Gram negatives	Gram positives	Yeast	Antimicrobial resistance markers
*A. calcoaceticus-baumannii complex* *Bacteroides fragilis* *Haemophilus influenzae* *Neisseria meningitidis* *Pseudomonas aeruginosa* *Stenotrophomonas maltophilia* *Enterobacterales spp.* *Enterobacter cloacae complex* *Escherichia coli* *Klebsiella aerogenes* *Klebsiella oxytoca* *Klebsiella pneumoniae group* *Proteus spp.* *Salmonella* *S. marcescens*	*Staphylococcus spp.* *Staphylococcus aureus* *Staphylococcus epidermidis* *Staphylococcus lugdunensis* *Streptococcus spp.* *Streptococcus agalactiae* *Streptococcus pyogenes* *Streptococcus pneumoniae* *Enterococcus faecalis* *Enterococcus faecium* *Listeria monocytogenes*	*Candida albicans* *Candida auris* *Candida glabrata* *Candida krusei* *Candida parapsilosis* *Candida tropicalis* *Cryptococcus neoformans/gattii*	*mecA/C* *mecA/C and MREJ (MRSA)* *van A/B* *blaCTX-M* *blaKPC* *blaIMP* *blaOXA-48* *blaNDM* *blaVIM* *mcr-1*

MRSA = Methicillin Resistant *Staphylococcus aureus*

Gold standard methods for pathogen identification included traditional culture-based biochemical techniques for phenotypic profiling (manual and automated), as well as the use of MALDI-TOF MS. The use of genotypic testing in the case of inconclusive or unclear results from conventional testing was also considered as an appropriate method to complement biochemical techniques or mass spectrometry when performed. Gold standard methods for the detection of antimicrobial resistance markers included phenotypic testing assessed by automated methods or by traditional phenotypic techniques, according to EUCAST and CLSI guidelines [16, 17]. Moreover, the genotypic confirmation of the antimicrobial resistance gene markers of interest on blood culture colonies, was also considered as an appropriate gold standard for comparison of resistance genes detected by the BCID2.

### Screening and selection of articles

Papers identified during the main database search strategy were exported into Endnote [18] and subsequently in Rayyan (https://www.rayyan.ai/) [19]. The screening of the papers was performed in Rayyan by two independent reviewers (A.M.P., W.L.), by screening title/abstract and the full text of the articles in one stage. The screening of the grey literature was performed by the two reviewers directly from the specific websites included in the search (see Additional file 1: Data 2).

Agreement between the two reviewers was assessed by the kappa coefficient. Discrepancies in the study selection were discussed after the screening; and if agreement could not be reached, a third reviewer was involved (L.F.K.).

### Data extraction

Data extracted included the number of true positives, false positives, false negatives, and true negatives, for each BCID2 target (Table 1), defined below, as well as time to pathogen identification and antimicrobial markers detection or AST availability according to the BCID2 and conventional culture-based methods.

For pathogen identification a true positive was defined as a result where the BCID2 panel and the gold standard comparator detected the same target organism. A false negative result was defined by the BCID2 failing to detect an organism target that was detected by the gold standard comparator, while a false positive result was defined by the BCID2 panel detecting an organism that was not detected by the gold standard methods. To be defined as a true negative result, the target organism should have not been detected by either method.

For antimicrobial resistance markers, a true positive was defined as a result where the BCID2 detected a specific resistance gene and the gold standard comparator showed a phenotypic resistant profile known to be associated with that genetic determinant and/or detected the genetic determinant of interest itself, by mean of standard genotypic testing performed on blood culture colonies. A false negative was defined as a result where the BCID2 did not detect a specific resistance gene and the gold standard culture-based comparator showed a phenotypic resistant profile normally induced by the genetic marker of interest and/or detected the presence of the gene of interest by mean of genotypic characterization on culture colonies. A false positive was defined as a result where the BCID2 detected a specific resistance gene, and the blood culture-based gold standard comparator showed a susceptible phenotype for those antimicrobials to which resistance is normally induced by the genetic determinant of interest and/or ruled out the presence of that resistance gene by mean of genotypic characterization on culture colonies. A true negative was defined as a result where the BCID2 did not detected a resistance marker of interest and the blood culture-based gold standard comparator showed a susceptible phenotype for those antimicrobials to which resistance is normally induced by the genetic determinant of interest and/or ruled out the resistance marker of interest by mean of genotypic characterization on culture colonies.

If both phenotypic and genotypic testing were performed by a study as gold standard comparators for assessing antimicrobial resistance and gave a discrepant result for a resistance profile of interest, the result from genotypic testing was chosen as gold standard comparator rather than the phenotypic result.

Of note, isolates showing intermediate resistance to specific antimicrobials at standard phenotypic testing were considered as resistant. Moreover, considering that the BCID2 panel no longer reports *mecA/C* for coagulase-negative staphylococci except for *S. epidermidis* and *S. lugdunensis*, accuracy of *mecA/C* was calculated for these two species only.

Blood cultures growing BCID2 off-panel pathogens were included in the analysis, while blood cultures resulting in BCID2 invalid runs were not included.

If information in the selected papers was unclear on incomplete, the authors were emailed for obtaining missing data. The manufacturer was also contacted to confirm whether any changes had been made from the “RUO prototype” versions to the RUO/IUO and IVD versions of the test.

### Quality assessment

The QUADAS-2 scale was used to assess the quality of studies included in the meta-analysis [20], whose main domains were adapted to our specific clinical question. Specifically, the domain “patient selection” was modified into “sample selection”; consistently, in all domains the word “patients” was always replaced by “blood culture samples”. Moreover, in the index test domain we did not include the signalling question about whether the threshold was pre-specified, as the test does not have any threshold. For the study reporting data about time to results only [21] the signalling questions about blinding in the index and reference standard domains of the risk of bias assessment were deemed not applicable, as well as the signalling question about genotypic confirmation in the reference standard domain of the applicability concerns section.

The quality scores were inputted in the Quality effects model as part of the sensitivity analysis for the main BCID2 targets.

### Statistical analyses

Contingency tables were built for each target of the BCID2, including bloodstream pathogens and resistance markers, by pooling results from monomicrobial and polymicrobial blood cultures, with the total number of samples for each table being equal to the number of blood cultures tested by each specific study.

Results were then assessed for specific targets and groups of targets of interest. The split component synthesis (SCS) method [22] using the inverse variance heterogeneity model [23] was utilised to estimate the pooled diagnostic odds ratio (DOR) and calculate the heterogeneity (I^2^). The SCS method then splits the DOR into its component parts, the logit sensitivity (Se) and logit specificity (Sp), and from there derives the negative and positive likelihood ratios (LR − and LR +). The area under the curve (AUC) was derived from the DOR using the following equation logit (AUC) = ln(DOR)/2.

Sensitivity analyses were conducted using the Quality effects model to assess the impact of the quality of the individual studies [24], the quality scores were taken from the quality assessment. Publication bias was assessed using of the Doi plot and the LFK index [25]. The funnel plot and existing tests for asymmetry (e.g. Egger, Begg) where not used as the standard errors of DOR could be misleading, and thus not suitable for meta-analyses of test accuracy [26]. The statistical analysis was performed with STATA/SE 16.1 using the *diagma* [27] and lfk modules [28].

Time to results were defined as the time from blood culture collection to pathogen identification and antimicrobial resistance markers detection or AST availability according to the BCID2 and conventional culture testing.

## Results

### Yield of search strategy

**Fig. 1:**
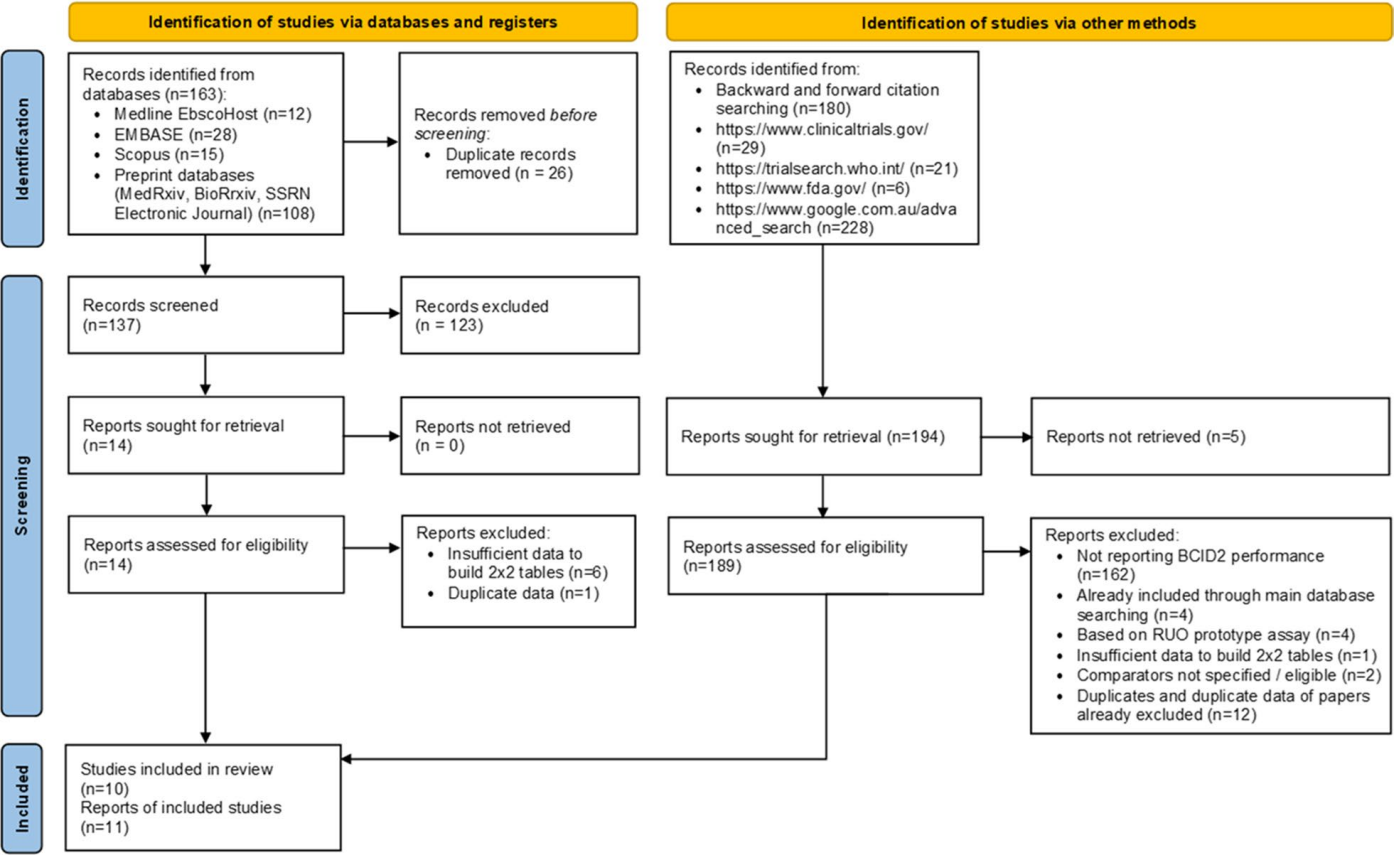
Study selection according to PRISMA 2020 flow diagram

The search strategy resulted in 163 papers and the backward & forward literature citation search performed in Scopus in 180 papers. The grey literature search identified further 284 records. As a result of the literature screening, ten studies met the inclusion criteria [9–14, 29]. Agreement between the two reviewer was substantial with a k correlation coefficient 0.7959 (95% CI 0.6178 to 0.9740). The PRISMA flow diagram reporting the steps of the study selection is shown in the Fig. 1 [30].

### Study characteristics

Eight out of ten included reports were published as original studies [9–14, 21, 29], one was a conference report [31], and one was a multicentric study whose results had both been published as a conference report as well as submitted by the manufacturer for obtaining FDA approval [32, 33].

Nine out of the ten selected studies reported data about the BCID2 accuracy in identifying pathogens and resistance genes markers compared to culture-based methods [8–14, 29, 31, 32]; of them two also reported data about time to results [12, 29]. One study reported data about time to results only [21].

Four of the selected studies were from Europe [9, 11, 13, 31], two from Australia [12, 29], one from the U.S [10], one from Hong Kong [14], one from India [21] and one was a multicentric study including sites both from the U.S. and Europe [32, 33]. Three out of ten studies were multicentric in design. All the 9 studies reporting data about the BCID2 accuracy for pathogens and resistance markers identification included fresh clinical blood culture samples [9–12, 29, 31–33], except one which included spiked samples only [14], and another one which assessed archived clinical samples [13]. Some studies including clinical samples, also included a subgroup of spiked blood cultures [9, 11]. One study included both data about clinical, archived and seeded samples but only the data from the clinical prospective group was included in our analysis as the other two groups were not eligible due to the comparator used [32, 33]. The number of samples assessed by each study ranged from 30 to 1074 and all studies included both monomicrobial and polymicrobial isolates. One study focused on paediatric blood cultures and three studies specifically on blood cultures collected from the Intensive Care Unit and/or Emergency Department. Table 2 summarizes the characteristics of the studies included in the metanalysis.

**Table 2:** Characteristics of studies included in the meta-analysis

Author, year	Study design	Country	Population	Type of samples (n)	Blood culture samples growth	Total samples^a^	Diagnostic accuracy	TAT
Berinson et al. 2021 [9]	Prospective, single centre	Germany	ICU, ED, adults	Clinical (182) + spiked (10)	Monomicrobial (161); polymicrobial (31)	192	Yes	No
Graff et al. 2021 [10]	Prospective, single centre	U.S	Any ward, paediatric	Clinical (191)	Monomicrobial (167); polymicrobial (24)	191	Yes	No
Holma et al. 2021 [11]	Prospective, single centre	Finland	Any ward	Clinical (102) + spiked (21)	Monomicrobial (71); polymicrobial (29); negative (23)	123	Yes	No
Sparks et al. 2021 [12]	Prospective, multi centre	Australia	Any ward	Clinical (49)	Monomicrobial (42); polymicrobial (7)	49	Yes	Yes
Cortazzo et al. 2021 [13]	Assessment of archived samples, single centre	Italy	Not specified	Archived (90)	Monomicrobial (55); polymicrobial (35)	90	Yes	No
Peri et al. 2022 [29]	Prospective, single centre	Australia	ICU, ED, adults	Clinical (62)	Monomicrobial (60); polymicrobial (2)	62	Yes	Yes
Sze et al. 2021 [14]	Assessment of seeded samples, single centre	Hong Kong	Not specified	Spiked (61)	Monomicrobial (46); polymicrobial (15)	61	Yes	No
Lu et al. 2019 [32, 33]	Prospective, multi centre	U.S., Europe	Not specified	Clinical (1074)	NA ^b^	1074	Yes	No
Camelena 2021 [31]	Prospective, multi centre	France	ICU, ED, haemato-oncology, surgery, adults and paediatrics	Clinical (163)	Monomicrobial (153); polymicrobial (10)	163	Yes	No
Shah et al. 2022 [21]	Prospective, single centre	India	ICU, adults and paediatrics	Clinical (30)	Monomicrobial (23); polymicrobial (7)	30	No	Yes

^a^Excluding invalid runs; ^b^125/1074 samples had multiple analytes detected by BCID2

TAT = Turnaround Time; ICU = Intensive Care Unit; ED = Emergency Department; NA = Not available

Two studies were funded by the manufacturer (either bioMérieux or Biofire diagnostics, which has currently been acquired by bioMérieux) [10, 32, 33], while in other cases the manufacturer supplied the laboratory kit consumables only [9, 12–14, 29]. In the study funded by Biofire diagnostics, it was disclosed that the funder had no role in study design, data collection and interpretation. In two cases funding was not disclosed [21, 31].

Pathogen identification was performed in 9/10 studies according to MALDI-TOF [9–14, 21, 29, 31], while different methods were used for AST according to local practices, including semi-automated methods (VITEK-2) and conventional phenotypic testing such as disk and gradient diffusion testing and broth microdilution in line with EUCAST and CLSI guidelines [16, 17]. Among the 9 studies reporting data to build 2 × 2 tables, genotypic confirmation of antimicrobial resistance genes in case of detection of phenotypic resistance was performed by all except one study [10]. One study reported data about pathogen identification and not about antimicrobial resistance markers detection [31]. Table 3 summarizes the conventional culture methods used as gold standard for pathogen identification and resistance detection by the different studies.

**Table 3:** Culture-based methods used by the different studies, serving as gold standard comparators for the BCID2

Study	Comparator for pathogen ID	Methods used for AST	Comparator for *blaCTX-M*	Comparator for carbapenemases	Comparator for *mcr-1*	Comparator for *mecA/C*	Comparator for *mecA/C & MREJ*	Comparator for *vanA/B*
Berinson et al. [9]	MALDI-TOF MS	VITEK-2	3rd gen. cephalosporin resistance at VITEK-2 confirmed by combination disk test; PCR^a^ if cephalosporin-R / BCID2-*blaCTX-M* negative isolates	Carbapenem resistance confirmed by PCR^b^	Not specified	Oxacillin resistance confirmed by PCR	Oxacillin resistance confirmed by immunochromatographic assay and GeneXpert SA/MRSA	Glycopeptide resistance confirmed by *vanA/B* PCR and GeneXpert *vanA-vanB*
Graff et al. [10]	MALDI-TOF MS	Phenotypic methods according to CLSI	3^rd^ gen. cephalosporin resistance	Carbapenem resistance	Phenotypic resistance	Oxacillin resistance	Oxacillin resistance	Glycopeptide resistance
Holma et al. [11]	MALDI-TOF MS confirmed by 16S rDNA seq if unclear results	Disk and/or MIC-gradient diffusion	Genotypic characterization	Genotypic characterization	Genotypic characterization	Genotypic characterization	Genotypic characterization	Genotypic characterization
Sparks et al. [12]	MALDI-TOF MS	VITEK-2 and/or disk diffusion, Etest	3rd gen. cephalosporin resistance + phenotypic confirmation of ESBL and/or PCR	Carbapenem resistance confirmed by PCR for *blaIMP*	Not specified	Oxacillin resistance confirmed by PCR	Oxacillin resistance confirmed by PCR	Glycopeptide resistance confirmed by PCR
Cortazzo et al. [13]	MALDI-TOF MS	PCR	PCR	PCR	PCR	PCR	PCR	PCR
Peri et al. [29]	MALDI-TOF MS	VITEK-2, disk diffusion, E-test	3rd gen. cephalosporin resistance confirmed by PCR. WGS in discordant cases	Carbapenem resistance	Not specified	Oxacillin resistance confirmed by PCR	Oxacillin resistance confirmed by PCR	Glycopeptide resistance
Sze et al. [14]	MALDI-TOF MS	BMD	3rd gen. cephalosporin resistance (disk test and BMD) confirmed by PCR	PCR	Colistin resistance confirmed by PCR	Oxacillin resistance confirmed by PCR	Oxacillin resistance confirmed by PCR	Glycopeptide resistance confirmed by PCR
Lu et al. [32, 33]	Standard manual and automated microbiological/biochemical methods	Manual and automated methods according to CLSI	Genotypic characterization	Genotypic characterization	Phenotypic resistance (BMD)	Genotypic characterization	Genotypic characterization	Genotypic characterization
Camelena^c^ [31]	MALDI-TOF MS	Not performed	-	-	-	-	-	-
Shah et al^d^ [21]	MALDI-TOF MS	VITEK-2	VITEK-2	VITEK-2	VITEK-2	VITEK-2	VITEK-2	VITEK-2

^a^For *blaCTX-M, blaTEM, and blaSHV*; ^b^For spiked samples: genotypic profile assessed by PCR and Whole Genome Sequencing; ^c^Data about pathogen identification only reported; ^d^Data about time to results only reported. ID = identification; MALDI-TOF MS = matrix-assisted laser desorption/ionization time of flight mass spectrometry; BMD = Broth Microdilution

Overall, the 9 studies reporting data about the BCID2 accuracy in identifying bloodstream pathogens and resistance markers compared to culture-based methods, included 2005 blood cultures assessed with both methods, after excluding BCID2 invalid runs (n = 3). Of these, 1913 were blood culture clinical samples (95.4%) and 92 (4.6%) spiked samples; moreover 268 (13.4%) yielded a polymicrobial result according to either conventional culture methods or the BCID2. Overall, 839 BCID2 on-panel Gram negative bacteria, 1159 Gram positives and 86 yeasts grew from the included samples. All BCID2 on-panel pathogens grew according to conventional methods on the blood cultures samples, including: *Enterobacterales* (n = 678), of which the most common species were *E. coli* (n = 354), *Klebsiella* spp. (n = 183), *E. cloacae* complex (n = 51), *Proteus* spp. (n = 31), *S. marcescens* (n = 25), and *Salmonella* (n = 24), non-fermenting bacilli (*P. aeruginosa* n = 73, *A. baumannii* n = 24, and *S. maltophila*, n = 21), *B. fragilis* (n = 24), Gram negative encapsulated bacteria (*N. meningitidis* n = 7, and *H. influenzae*, n = 12), *E. faecalis* (n = 79), *E. faecium* (n = 76), *L. monocytogenes* (n = 13), *Staphylococcus* spp*.* (n = 750), including *S. epidermidis* (n = 338), *S. aureus* (n = 238) and *S. lugdunensis* (n = 17), *Streptococcus* spp. (n = 241) and *Candida* spp. (n = 79). Among these isolates, the major resistance patterns detected according to conventional testing were: ESBL production in *Enterobacterales* (n = 114), resistance to carbapenems and/or production of carbapenemases in Gram-negatives (n = 59), resistance to oxacillin and/or genotypic detection of *mecA/C* (n = 212) and *mecA/C* & *MREJ* in *Staphylococcus* spp*.* (n = 76) and resistance to vancomycin and/or detection of *vanA/B* in *Enterococcus* spp. (n = 44). Resistance to colistin was rarely tested in carbapenem susceptible isolates as per local practices, and colistin isolates were rarely detected (n = 18).

### Time to results

Three studies only reported the time from blood culture collection to results according to the BCID2 and conventional culture methods [12, 21, 29], including time to pathogen identification and time to resistance markers detection and/or AST.

Specifically, according to Sparks et al. [12] mean time (± standard deviation [SD]) from blood culture collection to pathogen identification with the BCID2 and conventional culture methods were 24.6 (± 16.8) h and 38.32 (± 21.9) h respectively; moreover, mean time (± SD) to resistance detection for blood cultures containing bla_CTX-M_ alleles with the BCID2 was 21.3 (± 0.4) h while mean time (± SD) to culture-based PCR and conventional AST for the same samples was 50.7 (± 0.4) h. A second study [29] estimated that if the BCID2 had been implemented in the clinical workflow, pathogen identification and resistance markers detection would have been available 9.69 h (95% CI: 7.85 to 11.53) and 27.8 h (95% CI: 23.05 to 32.55) sooner compared to conventional testing. However, these were only estimated times as the BCID2 was not run real time.

According to a third study median time to BCID2 results was 21 h while median times to pathogen identification and AST according to culture-based methods were 42 and 49 h respectively [21].

A fourth study [10] reported time to results according to the BCID2 but not according to conventional culture methods, and the remaining studies did not report any time to results. Therefore, overall, data about time to results were not considered enough to be assessed in the meta-analysis.

### Quality assessment

Low quality assessment in the sample selection domain was mainly due to the exclusion by some studies (2/9, 22%) of some blood culture samples based on the time to positivity [9, 14] as well as to the lack of disclosure of whether consecutive samples were included (7/9 studies, 78%) [11–14, 29, 31–33], despite case control design was always avoided. Moreover, in the index and gold standard test domains, it was unclear for some studies (7/9, 78%)[9, 11, 13, 14, 29, 31–33] whether the index test interpreter was blind to the results of the gold standard and vice versa (5/9 studies, 56%)[9–11, 31–33]. Overall, the main reason for high risk of bias was found in the flow and timing domain and was due to the use by most studies (5/9, 56%) of different gold standards on different samples, mainly when performing PCR sequencing only on phenotypically resistant isolates [9, 12, 14, 29]. Nonetheless, most studies (5/9, 56%) reported a good score relatively to all the applicability domains (see Additional file 1: Data 3a and 3b).

### Performance of BCID2

**Fig. 2:**
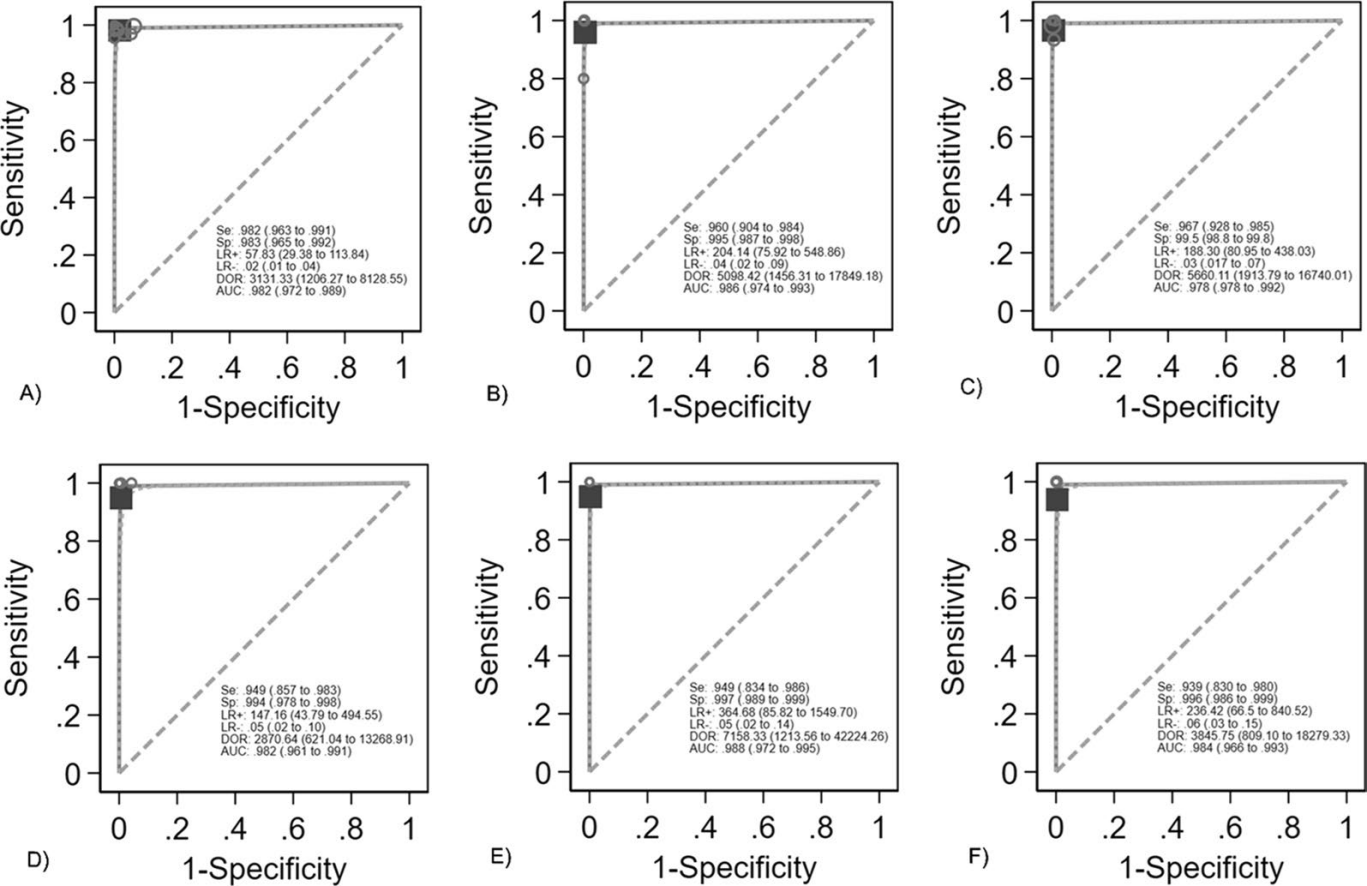
BCID2 ROC-curves: **A** *Enterobacterales* **B** *S. aureus* **C** *Streptococcus* **D** *bla*_CTX-M_ **E** carbapenemases **F** *mecA/C&MREJ*. Weights are from Doi’s IVhet model the Curve. ROC = Receiver operating characteristic; Se = Sensitivity; Sp = Specificity; LR = Likelihood Ratio; DOR = Diagnostic Odds Ratio; AUC = Area Under the Curve

Figure 2a–f and Additional file 1: Data 4 summarize the performance of the assay for the most relevant on-panel determinants of bloodstream infection, including *Enterobacterales, S. aureus, Streptococcus* spp., *bla*_CTX-M_, carbapenemases and *mecA/C & MREJ* [34, 35].

Overall, the BCID2 panel showed good performances for the detection of such targets. Specifically, the pooled sensitivity and specificity of the BCID2 for the detection of *Enterobacterales* were 98.2% (95% CI 96.3 to 99.1) and 98.3% (95% CI 96.5 to 99.2) respectively, with an Area Under the Curve (AUC) of 0.982 (95% CI 0.972 to 0.989) (Fig. 2a). Of note, among 58 false positives results for *Enterobacterales* detected in all studies, 53 were attributed to the presence of nucleic acid from non-viable *E. coli* in specific lots of blood culture bottles in the multicentric pivotal study [32, 33]. For *S. aureus* sensitivity and specificity were 96.0% (95% CI 90.4 to 98.4) and 99.5% (95% CI 98.7 to 99.8) respectively, with an AUC of 0.986 (95% CI 0.974 to 0.993) (Fig. 2b) and for *Streptococcus* spp. sensitivity was 96.7% (95% CI 92.8 to 98.5), specificity 99.5% (98.8 to 99.8) and the AUC 0.978 (95% CI 0.978 to 0.992) (Fig. 2c). The performance of the BCID2 for the detection of the main on-panel antimicrobial resistance markers was also high, with a pooled sensitivity and specificity of 94.9% (95% CI 85.7 to 98.3) and 99.4% (95% CI 97.8 to 99.8) for *bla*_CTX-M_, 94.9% (95% CI 83.4 to 98.6) and 99.7% (95% CI 98.9 to 99.9) for carbapenemases, and 93.9% (95% CI 83.0 to 98.0) and 99.6% (95% CI 98.6–99.9) for mecA/C & MREJ (Fig. 2d-e).

Table 4 summarizes the performance of the assay for the other BCID2 targets or subgroups of targets of interest. Overall, specificity was above 98% for all targets or subgroups of targets assessed, with a narrow confidence interval. Few false positive results were reported, mainly for *S. epidermidis,* where the lower limit of the confidence interval for specificity was 93.1%, and for *mecA/C* [9, 32, 33]. Of note, in one of the 2 studies were most false positive results were reported, *S. epidermidis* was not unfrequently detected by the BCID2 panel in place of other coagulase-negative staphylococci [9].

**Table 4:** Pooled performance characteristics of the BCID2 for specific targets and sub-groups of targets of interest

	SE% (95% CI)	SP% (95% CI)	LR + (95% CI)	LR- (95% CI)	DOR (95% CI)	AUC (95% CI)	Studies included	*I*^2^
*E. coli*	98.1 (95.5–99.2)	99.5 (98.7–99.8)	179.15 (74.98–428.05)	0.02 (0.01–0.04)	9343.81 (2940/24–29,693.80)	0.990 (0.982–0.994)	9	0
*Klebsiella spp.*^*a*^	95.4 (89.6–98.0)	99.5 (98.7–99.8)	184.85 (72.68–470.13)	0.05 (0.02–0.10)	3979.15 (1227.25–12,901.71	0.984 (0.972–0.991)	9	0
*Proteus spp.*	85.0 (68.0–93.8)	99.6 (98.7–99.9)	232.04 (66.15–813.96)	0.15 (0.07–0.32)	1540.46 (358.89–6612.17)	0.975 (0.950–0.988)	7	0
*Salmonella*	85.0 (66.6–94.1)	99.7 (98.9–99.9)	289.25 (75.46–1108.74)	0.15 (0.07–0.33)	1917.95 (406.35–9052.75)	0.978 (0.953–0.990)	7	0
*E. cloacae and S. marcescens*	91.6 (79.6–96.8)	99.7 (98.9–99.9)	262.42 (79.55–865.68)	0.09 (0.04–0.20)	3101.8 (739.35–13,013.06)	0.982 (0.965–0.991)	8	6.6
*A. baumannii complex*	87.4 (69.0–95.6)	99.8 (99.0–99.9)	350.49 (84.19–1459.07)	0.13 (0.05–0.29)	2780.81 (529.13–14,614.38)	0.981 (0.958–0.992)	5	0
*P. aeruginosa*	92.7 (83.1–97.0)	99.6 (98.8–99.9)	215.16 (73.83–627.00)	0.07 (0.04–0.20)	2930.15 (795.44–10,793.74)	0.982 (0.966–0.990)	9	0
*S. maltophilia*	86.1 (63.0–95.8)	99.8 (99.0–1.00)	454.15 (86.29–2390.39)	0.14 (0.05–0.36)	3270.71 (484.31–22,088.03)	0.983 (0.957–0.993)	4	0
*B. fragilis*	87.2 (70.4–95.1)	99.5 (98.2–99.9)	173.12 (47.72–628.13)	0.13 (0.06–0.29)	1340.50 (292.22–6149.32)	0.973 (0.945–0.987)	6	0
*H. influenzae*	78.7 (39.9–95.3)	99.8 (98.1–1.00)	462.98 (41.17–5206.10)	0.21 (0.06–0.72)	2167.39 (144.62–32,481.75)	0.979 (0.923–0.994)	3	31.9
*N. meningitidis*	73.1 (39.1–92.0)	99.8 (98.1–1.00)	327.67 (38.27–2805.62)	0.27 (0.10–0.75)	1213.69 (112.58–13,083.73)	0.972 (0.914–0.991)	3	0
*E. faecalis*	92.3 (83.5–96.6)	99.6 (98.9–99.8)	225.77 (83.73–608.76)	0.08 (0.04–0.15)	2938.91 (881.60–9797.14)	0.982 (0.967–0.990)	9	0
*E. faecium*	92.3 (81.9–97.0)	99.6 (98.7–99.9)	216.44 (70.69–662.76)	0.08 (0.04–0.17)	2816.75 (722.86–10,975.99)	0.982 (0.964–0.991)	7	0
*L. monocytogenes*	86.1 (55.7–96.8)	99.9 (98.9–1.00)	577.99 (73.61–4538.35)	0.14 (0.04–0.44)	4157.81 (393.01–43,987.07)	0.985 (0.952–0.995)	3	0
*Staphylococcus spp.*	97.4 (94.1–98.8)	98.8 (97.3–99.5)	83.35 (36.93–188.14)	0.03 (0.13–0.06)	3133.31 (1044.73–9397.36)	0.982 (0.970–0.990)	9	17.4
*S. epidermidis*	92.0 (77.4–97.5)	98.1 (93.1–99.5)	48.79 (13.35–178.29)	0.08 (0.03–0.23)	600.80 (113.95–3167.703)	0.961 (0.914–0.983)	8	52.7
*S. lugdunensis*	87.7 (66.1–96.3)	99.6 (98.0–99.9)	211.065 (42.892–1038.63)	0.12 (0.05–0.34)	1708.12 (260.86–11,184.63)	0.976 (0.942–0.991)	4	0
*Candida spp.*^b^	92.0 (81.5–96.8)	99.5 (98.6–99.8)	199.18 (66.57–595.94)	0.08 (0.04–0.17)	2474.911 (653.62–9371.24)	0.980 (0.962–0.990)	8	0
*mcr-1*	77.3 (42.6–94.0)	99.6 (96.4–1.00)	183.42 (21.61–1556.53)	0.23 (0.07–0.71)	804.36 (71.33–9071.12)	0.966 (0.894 to0.990)	3	0
*mecA/C*	93.8 (88.6–96.7)	98.8 (97.6–99.4)	78.94 (38.88–160.24)	0.06 (0.04–0.11)	1262.31 (510.55–3121.01)	0.973 (0.958–0.982)	8	0
*vanA/B*	90.9 (76.7–96.8)	99.7 (99.0–99.9)	347.03 (92.35–1304.12)	0.09 (0.04–0.21)	3820.95 (793.29–18,403.99)	0.984 (0.966–0.993)	5	0

SE = Sensitivity; SP = Specificity; LR = Likelihood Ratio; DOR = Diagnostic Odds Ratio; AUC = Area Under the Curve. ^a^Including* K. aerogenes, K. oxytoca and K. pneumoniae group;* ^b^Including *C. albicans, C. auris, C. glabrata, C. cruzei, C. parapsilosis, C. tropicalis*

In regard to pooled sensitivity, this was overall inversely proportional to the frequency the targets or subgroups of targets were detected in the whole samples’ population. Specifically, sensitivity was > 95% for *E. coli* and *Staphylococcus* spp. Moreover, for other targets commonly detected in the setting of bloodstream infections (including *Klebsiella* spp., enterococci, *P. aeruginosa, Candida* spp., and *mecA/C*) sensitivity values where > 90%, with the lower limit of confidence intervals > 80% (slightly lower for *S. epidermidis* and *vanA/B*). Other relevant but less common pathogens (such as *Proteus* spp*., Salmonella, A. baumanni, S. maltophila, B. fragilis* and *S. lugdunensis*) maintained a sensitivity > 80% (with 95% CI > 60%), while for rarer targets (such as *H. influenzae, N. meningitidis, L. monocytogenes,* and *mcr-1*) sensitivity ranged from 73 to 86% with the lower limit of confidence intervals between 39 and 55%.

The AUC of the targets and subgroups of targets of interest were > 96% in most cases, with narrow confidence intervals.

The rate of invalid results reported by the studies was low as 3 samples only were excluded due to this reason.

Heterogeneity was absent for all the targets and subgroups of targets considered except for *S. epidermidis*, where a moderate heterogeneity was found (52.7%), likely due to the relatively high number of false positives detected by some studies compared to others, as discussed [9, 32, 33], and for *H. influenzae,* where a low heterogeneity was found (31.9%) (Table 4).

The Doi plots for *Enterobacterales, S. aureus, Streptococcus* spp, *bla*_CTX-M_ and carbapenemases showed no asymmetry, and for *mecA/C & MREJ* minor asymmetry was found, overall excluding publication bias for the 6 major determinants of bloodstream infection (Additional file 1: data 5). Among the other targets assessed, major asymmetry of the Doi plots favouring studies with higher discretionary capacity suggesting the presence of publication bias was observed for *B. fragilis* (LFK index = 2.82), *S. epidermidis* (LFK index = 3.19) and *mecA/C* (LFK index -2.29) (data not shown). This finding is not surprising considering some studies report a slightly less accurate performance of these targets compared to others [9, 32, 33].

The sensitivity analyses for the 6 main determinants of bloodstream infection (*Enterobacterales, S. aureus, Streptococcus* spp., *bla*_CTX-M_, carbapenemases and mecA/C & MREJ) using the Quality effects models are shown in the Additional file 1: data 6 and confirm a good performance of the assay.

Five out of nine studies reported data about concordance between culture-based methods and BCID2 on polymicrobial blood cultures samples (see Table 5). Overall, full concordance between the 2 methods was 75/90 (83%) polymicrobial samples, including pathogen identification and resistance marker detection.

**Table 5:** Agreement between culture-based methods and BCID2 on polymicrobial blood culture samples

Study	Concordant results / total polymicrobial samples (%)
Berinson et al. [9]	19/31 (61.3)
Sparks et al. [12]	5/7 (71.4)
Cortazzo et al. [13]	35/35 (100)
Peri et al. [29]	1/2 (50)
Sze et al. [14]	15/15 (100)
Total	75/90 (83)

In the study by Lu et al. [32, 33] 125/1074 positive blood cultures samples had multiple targets detected by BCID2 of which 43/125 (34.4%) had perfect agreement with conventional testing. When omitting the 53 false positive results due to the presence of nucleic acid from non-viable *E. coli* in specific lots of blood culture bottles, agreement with conventional testing was 43/84 (51.2%)

686 new BCID2 targets (not included in the previous version of the test) were detected in 1913 clinical blood culture samples. Of those 335 were *S. epidermidis* and 112 antimicrobial resistance markers*.*

## Discussion

The results of our meta-analysis confirm overall a good performance of the BCID2 in detecting bloodstream pathogens and associated resistance markers.

In particular, the specificity of the assay was excellent across all targets investigated. Regarding the BCID2 sensitivity, this was high (> 95%) for targets which have a major role in the aetiology of bloodstream infection, including *Enterobacterales, S. aureus, Streptococcus* spp., *P. aeruginosa,* enterococci and *Candida* spp., as well as some major resistance determinants such as *bla*_CTX-M_, carbapenemases and *mecA/C* and *MREJ* (> 90% with 95% CI ranging from 80 to 99%). In settings like sepsis, where early appropriate antimicrobial treatment is associated with survival [36], these performance values my help to optimise early patient management improving clinical outcomes.

Nonetheless, overall, few studies were included in our meta-analysis, due to the recent release of the BCID2 panel, partially preventing a robust assessment of the sensitivity of those pathogens or resistance markers which are less common in the setting of bloodstream infections.

The ten selected studies did not report enough information about turnaround times to be assessed in the meta-analysis. Available data suggest that the BCID2 can identify bloodstream pathogens and some antimicrobial resistance markers up to 10 h and 28 h earlier than conventional culture-based testing respectively, although this may vary according to local practices (i.e., use of MALDI-TOF on early subcultures) and further studies are needed to better define times to result compared to traditional techniques. Yet, the turnaround time of the BCID2 from a positive blood culture is well defined (1 h), and each centre could easily estimate the potential time gained with the implementation of the test based on their laboratory workflow and local practices.

Reduced turnaround time, however, is not sufficient to indicate the utility of a diagnostic test for bloodstream infection, and several additional factors should be considered. Among these, the possibility of running the test 24/7 rather than during business hours, based on the resources of the clinical laboratory, is likely going to affect the impact of the test on timely antimicrobial prescriptions. Moreover, despite the scarcity of randomized controlled trial in this field, a recent meta-analysis has shown that the impact of rapid diagnostic tests for bloodstream infection on patients’ outcome is dependent on the real-time implementation of the tests’ results by mean of antimicrobial stewardship programs [37]. Several rapid tests for the diagnosis of bloodstream infection have become available in the recent years, and the advantages of implementing one test over the other in a specific setting should also take into account the local epidemiology of antimicrobial resistance, as well as health economic endpoints. As compared to the previous version of the test, in our meta-analysis the BCID2 was able to detect up to 686 of the new targets (of which 335 where *S. epidermidis*) in 1913 clinical blood culture samples, likely proving an increased diagnostic usefulness compared to the prior panel.

Our study has some limitations that should be acknowledged. The first limitation is the lack of assessment of polymicrobial BC as a separate group from monomicrobial samples. Some studies reported how the agreement of the BCID2 results with conventional methods was lower for polymicrobial than monomicrobial samples [9, 12, 29], and this was not assessed in our analysis by means of diagnostic accuracy measures, since 2 × 2 tables could not be calculated for specific targets on polymicrobial samples. Specifically, Berinson et al. reported a concordance for polymicrobial samples of 63% (19/31) with the most common discrepancies due to the misidentification of coagulase-negative staphylococci species or additional growth of coagulase-negative staphylococci undetected by the BCID2[9]. Moreover, in the study from Sparks et al. 2/7 polymicrobial samples had discordant results between the two methods, although in one case the additional growth observed according to conventional testing was thought to be due to a laboratory contamination, and the second discrepant case consisted in the lack of detection of *K. oxytoca* on a sample where *K. pneumoniae* also grew and was appropriately identified [12]. In a third study 1 out of 2 polymicrobial sample was found to have discordant results according to the two methods but whole genome sequencing confirmed results from the BCID2 rather than those from conventional culture [29]. Lastly, in the multicentric pivotal study 125/1074 positive blood cultures samples had multiple targets detected by BCID2 of which 43/125 (34.4%) had perfect agreement with conventional testing; notably, when omitting the 53 false positive results due to the presence of nucleic acid from non-viable *E. coli* in specific lots of blood culture bottles, agreement with conventional testing was 43/84 (51.2%).

Differently, other studies reported an agreement of 100% on polymicrobial samples.

The high number of false positives detected in the pivotal study due to the presence of non-viable *E. coli* in some blood cultures bottles highlights a limitation of tests based on the detection of pathogen DNA rather than conventional cultures, which can falsely detect non-viable pathogens.

Another limitation of our meta-analysis relates to our definition of the “I” category as “intermediate resistance”. Current EUCAST guidelines now use the definition of “I” as “susceptible, increased exposure” [38], rather than as “intermediate”, as we have done, following the approach used by the papers we have included. As such, our classification of isolates into resistant (including the previously defined intermediate isolates) or susceptible should be interpreted according to the former guidelines. Overall, very few isolates were classified as “intermediate” (n = 7).

A third limitation of our meta-analysis is the heterogeneity of the microbiological methods used as gold standard comparators for the detection of antimicrobial resistance, which included both phenotypic and genotypic testing. Overall, in one study only [10] antimicrobial resistance was assessed by means of phenotypic methods only, not accompanied by any genotypic confirmation, with, however, no major discrepancies observed with the BCID2 results. However, it should be acknowledged that most of the other studies used the genotypic characterization only to confirm a resistant phenotype detected by conventional culture methods or an antimicrobial resistance marker detected by the BCID2, or to investigate discordant results between phenotypic AST and BCID2 [9, 12, 14, 29], without performing a blind genotypic assessment of all samples. Given resistance genes can sometimes be expressed at clinically insignificant levels, the lack of an extended genotypic characterization of all samples might have affected the assessment of the performance of the BCID2 for antimicrobial markers detection.

Nevertheless, it must be acknowledged that the interaction between genotypic and phenotypic resistance is complex [39], and the use of molecular methods for the detection of resistance markers in the setting of clinical infections always requires critical thinking, both in the case of negative results (where phenotypic resistance might still be possible due to underlying different genetic determinants) and positive ones (where the genes detected might not be expressed, leading to a susceptible phenotype). Moreover, in the case of the BCID2, while for some genetic markers the association with the phenotypic resistance is univocal and very well established (i.e., *mecA* for oxacillin resistance in *Staphylococcus* spp.) [40], in other cases, the on-panel genetic markers only explain a fraction of a specific phenotypic profile. This is for example the case of the detection of *bla*_CTX-M_, which cannot itself explain all the cases of 3^rd^ generation cephalosporin resistance, whose genotypic determinants are more numerous within the setting of the Extended Spectrum Beta Lactamases and AmpC enzymes [41]. Moreover, as known, the BCID2 is not able to detect all CTX-M variants (i.e., CTX-M-151) although it does detect the majority of them [8].

The accuracy of an assay in terms of sensitivity and specificity lacks the capacity to capture all these complex scenarios, which are extremely relevant from the clinical point of view. Diagnostic accuracy is an important component in the assessment of the utility of a rapid diagnostic test but should be complemented by an evaluation of its real-life impact in clinical practice, incorporating the confidence of acting upon the results provided. This might best be performed by undertaking a cluster randomised trial in which laboratories are randomly assigned to use of BCID2 or conventional methods. The impact on clinically relevant outcomes associated to the use of the system combined with appropriate antimicrobial stewardship could then be determined.

## Conclusions

The BCID2 showed good performance for the detection of on-panel targets including bloodstream pathogens and antimicrobial resistance determinants, making the assay a promising tool to implement in clinical practice. Nonetheless, in the clinical scenario, the use of molecular methods for the detection of antimicrobial resistance should always be interpreted carefully when used for clinical decision making, due to the complex interaction between genetic determinants and phenotypic profiles.

## Supplementary Information

**Additional file 1.** Supplementary data.

## Data Availability

Not applicable.

## Abbreviations

BCID2: BioFire blood culture identification 2 panel
MALDI-TOF MS: Matrix-assisted laser desorption/ionization time of flight mass spectrometry
AST: Antimicrobial susceptibility testing
SE: Sensitivity
SP: Specificity
PRISMA-DTA: Preferred Reporting Items for Systematic Reviews and Meta-Analyses of Diagnostic Test Accuracy Studies
CE-IVD: Conformité Européenne In vitro Diagnostics
FDA: Food and drug administration
TGA: Therapeutic goods administration
RUO: Research use only (RUO)
IUO: Investigational use only
SCS: Split component synthesis
DOR: Diagnostic odds ratio
LR: Likelihood ratios
AUC: Area under the curve
CI: Confidence interval
SD: Standard deviation
