# Supplementary material for: Performance of BioFire Blood Culture Identification 2 Panel (BCID2) for the detection of bloodstream pathogens and their associated resistance markers: a systematic review and meta-analysis of diagnostic test accuracy studies
Source: BMC Infect Dis. 2022 Oct 20;22:794. doi: 10.1186/s12879-022-07772-x (PMC9585790; doi:10.1186/s12879-022-07772-x)
Supplement: Supplementary file 1 — Additional file 1. Supplementary data. [file 12879_2022_7772_MOESM1_ESM.docx]

**Supplementary data 1. PRISMA-DTA Checklist**

| **Section/topic** | **#** | **PRISMA-DTA Checklist Item** | **Reported on page #** |
| --- | --- | --- | --- |
| **TITLE / ABSTRACT** | | |  |
| Title | 1 | Identify the report as a systematic review (+/- meta-analysis) of diagnostic test accuracy (DTA) studies. | 5 |
| Abstract | 2 | Abstract: See PRISMA-DTA for abstracts. |  |
| **INTRODUCTION** | | |  |
| Rationale | 3 | Describe the rationale for the review in the context of what is already known. | 5 |
| Clinical role of index test | D1 | State the scientific and clinical background, including the intended use and clinical role of the index test, and if applicable, the rationale for minimally acceptable test accuracy (or minimum difference in accuracy for comparative design). | 4 |
| Objectives | 4 | Provide an explicit statement of question(s) being addressed in terms of participants, index test(s), and target condition(s). | 5 |
| **METHODS** | | |  |
| Protocol and registration | 5 | Indicate if a review protocol exists, if and where it can be accessed (e.g., Web address), and, if available, provide registration information including registration number. | NA |
| Eligibility criteria | 6 | Specify study characteristics (participants, setting, index test(s), reference standard(s), target condition(s), and study design) and report characteristics (e.g., years considered, language, publication status) used as criteria for eligibility, giving rationale. | 6, 7 |
| Information sources | 7 | Describe all information sources (e.g., databases with dates of coverage, contact with study authors to identify additional studies) in the search and date last searched. | 5, 6 |
| Search | 8 | Present full search strategies for all electronic databases and other sources searched, including any limits used, such that they could be repeated. | Suppl 2 |
| Study selection | 9 | State the process for selecting studies (i.e., screening, eligibility, included in systematic review, and, if applicable, included in the meta-analysis). | 7 |
| Data collection process | 10 | Describe method of data extraction from reports (e.g., piloted forms, independently, in duplicate) and any processes for obtaining and confirming data from investigators. | 7 |
| Definitions for data extraction | 11 | Provide definitions used in data extraction and classifications of target condition(s), index test(s), reference standard(s) and other characteristics (e.g. study design, clinical setting). | 7, 8 |
| Risk of bias and applicability | 12 | Describe methods used for assessing risk of bias in individual studies and concerns regarding the applicability to the review question. | 9, Suppl 3a |
| Diagnostic accuracy measures | 13 | State the principal diagnostic accuracy measure(s) reported (e.g. sensitivity, specificity) and state the unit of assessment (e.g. per-patient, per-lesion). | 9, 10 |
| Synthesis of results | 14 | Describe methods of handling data, combining results of studies and describing variability between studies. This could include, but is not limited to: a) handling of multiple definitions of target condition. b) handling of multiple thresholds of test positivity, c) handling multiple index test readers, d) handling of indeterminate test results, e) grouping and comparing tests, f) handling of different reference standards | 8, 9 |
| Meta-analysis | D2 | Report the statistical methods used for meta-analyses, if performed. | 9, 10 |
| Additional analyses | 16 | Describe methods of additional analyses (e.g., sensitivity or subgroup analyses, meta-regression), if done, indicating which were pre-specified. | NA |
| **RESULTS** | | |  |
| Study selection | 17 | Provide numbers of studies screened, assessed for eligibility, included in the review (and included in meta-analysis, if applicable) with reasons for exclusions at each stage, ideally with a flow diagram. | 10, Fig 1 |
| Study characteristics | 18 | For each included study provide citations and present key characteristics including: a) participant characteristics (presentation, prior testing), b) clinical setting, c) study design, d) target condition definition, e) index test, f) reference standard, g) sample size, h) funding sources | 10, 11, 12  Table 2 & 3 |
| Risk of bias and applicability | 19 | Present evaluation of risk of bias and concerns regarding applicability for each study. | 13, 14  Suppl 3b |
| Results of individual studies | 20 | For each analysis in each study (e.g. unique combination of index test, reference standard, and positivity threshold) report 2x2 data (TP, FP, FN, TN) with estimates of diagnostic accuracy and confidence intervals, ideally with a forest or receiver operator characteristic (ROC) plot. | Suppl 4 |
| Synthesis of results | 21 | Describe test accuracy, including variability; if meta-analysis was done, include results and confidence intervals. | 14, 15,  Fig 2, Table 4, Suppl 4 & 6 |
| Additional analysis | 23 | Give results of additional analyses, if done (e.g., sensitivity or subgroup analyses, meta-regression; analysis of index test: failure rates, proportion of inconclusive results, adverse events). | NA |
| **DISCUSSION** | | |  |
| Summary of evidence | 24 | Summarize the main findings including the strength of evidence. | 16, 17 |
| Limitations | 25 | Discuss limitations from included studies (e.g. risk of bias and concerns regarding applicability) and from the review process (e.g. incomplete retrieval of identified research). | 17, 18 |
| Conclusions | 26 | Provide a general interpretation of the results in the context of other evidence. Discuss implications for future research and clinical practice (e.g. the intended use and clinical role of the index test). | 19 |
| **FUNDING** | | |  |
| Funding | 27 | For the systematic review, describe the sources of funding and other support and the role of the funders. | 20 |

**Supplementary data 2. Details of the search strategy**

**Medline EbscoHost -** (BCID W1 2) OR BCID2   OR “BIOFIRE Blood Culture Identification 2” OR “BioFire FilmArray Blood Culture Identification 2” OR (“Blood culture Identification” W1 “panel 2”) OR (“Biofire Filmarray” W1 “panel 2”) OR “Blood culture Identification panel 2”

**Embase -** ‘BCID 2’ OR BCID2   OR ‘BIOFIRE Blood Culture Identification 2’ OR ‘BioFire FilmArray Blood Culture Identification 2’ OR (‘Blood culture Identification’ AND ‘panel 2’) OR (‘Biofire Filmarray’ AND ‘panel 2’) OR ‘Blood culture Identification panel 2’

**Scopus -** TITLE-ABS ( ( bcid  W/1  2 )  OR  bcid2  OR  "BIOFIRE Blood Culture Identification 2"  OR  "BioFire FilmArray Blood Culture Identification 2"  OR  ( "Blood culture Identification"  W/1  "panel 2" )  OR  ( "Biofire Filmarray"  W/1  "panel 2" )  OR  "Blood culture Identification panel 2" )

**Pre-print databases**

**MedRxiv, BioRrxiv -** Biofire Blood Culture Identification

**SSRN Electronic Journal:** Biofire; Filmarray

**Grey literature**

https://www.clinicaltrials.gov/ - "Biofire Filmarray"

https://trialsearch.who.int/ - "Biofire Filmarray"

https://www.fda.gov/ - "BCID2"

https://www.google.com.au/advanced_search - "BCID2” filetype:pdf

**Supplementary data 3a. QUADAS-2 tool used for assessment of the quality of included studies**

|  | **Risk of bias** |  |  | **Applicability** |  |  |
| --- | --- | --- | --- | --- | --- | --- |
| **Domains** | **Signalling question** | **Yes** | **No*** | **Question** | **Low concern** | **High concern*** |
| **1. Sample selection** |  |  |  |  |  |  |
|  | **Was a consecutive or random sample of positive blood cultures included?** | *The study included a consecutive or random sample of positive blood cultures* | *The study did not include a consecutive or random sample of positive blood cultures* | **Is there concern that the included blood culture samples do not match the review question?** | *The blood culture samples were representative of the common bloodstream pathogens and associated AMR* | *The blood culture samples were not representative of the common bloodstream pathogens and associated AMR* |
|  | **Was a case-control design avoided?** | *The study avoided a case-control design* | *The study used a case-control design* |  |  |  |
|  | **Did the study avoid inappropriate exclusions?** | *The study avoided inappropriate exclusion (i.e., based on flag times or polymicrobial samples)* | *The study did not avoid inappropriate exclusion (i.e., based on flag times or polymicrobial samples)* |  |  |  |
| **2. Index test** |  |  |  |  |  |  |
|  | **Were the index test results interpreted without knowledge of the results of the gold standard?** | *The interpreter of index test results was blind to the gold standard test results* | *Index test results were interpreted with knowledge of the gold standard test results* | **Is there concern that the index test, its conduct, or interpretation differ from the review question?** | *The BCID2 panel was performed according to the manufacturer instructions* | *The BCID2 panel was not performed according to the manufacturer instructions* |
| **3. Gold standard** |  |  |  |  |  |  |
|  | **Is the gold standard likely to correctly classify the target condition?** | *For pathogen ID the gold standard is a traditional culture-based phenotypic method or MALDI-TOF MS. For AMR the gold standard is a phenotypic method according to EUCAST / CLSI or a genotypic method on culture colonies* | *The gold standard is not a phenotypic method according to EUCAST / CLSI nor MALDI-TOF MS nor a genotypic method applied on culture colonies (for AMR detection)* | **Is there concern that the target condition as defined by the reference standard does not match the review question?** | *The study confirmed AMR detected with phenotypic testing with a genotypic method* | *The study assessed AMR by means of phenotypic testing only, without genotypic confirmation* |
|  | **Were the gold standard results interpreted without knowledge of the index test results?** | *The interpreter of the gold standard test results was blind to the index results* | *Gold standard test results were interpreted with knowledge of the index test results* |  |  |  |
| **4. Flow & timing** |  |  |  |  |  |  |
|  | **Was there an appropriate interval between index test(s) and gold standard?** | *The index test and the gold standard were performed on the same sample* | *The index test and the gold standard were performed on different samples (i.e., from the same patient)* |  |  |  |
|  | **Did samples receive the same gold standard?** | *The study used the same gold standard for all blood culture samples* | *The study did not use the same gold standard test for all blood culture samples* |  |  |  |
|  | **Were all samples included in the analysis?** | *All blood cultures samples analysed with both methods were included in the analysis* | *Not all blood cultures samples analysed were included in the analysis* |  |  |  |

*AMR = Antimicrobial Resistance; BCID2 = Blood Culture Identification 2; ID = identification; EUCAST = European Committee on Antimicrobial Susceptibility Testing; CLSI = Clinical & Laboratory Standards Institute; MALDI-TOF MS = Matrix-Assisted Laser Desorption/Ionization Time Of Flight Mass Spectrometry*

* *The answer to the signalling questions was “unclear” if the study did not provide enough information to answer the question*

**Supplementary data 3b. Quality assessment of studies included in the meta-analysis according to the Quadas-2 tool**

| **Study** | **Risk of bias** | | | | **Applicability concerns** | | | **Quality Score** |
| --- | --- | --- | --- | --- | --- | --- | --- | --- |
|  | **Sample selection** | **Index test** | **Reference standard** | **Flow and timing** | **Sample selection** | **Index test** | **Reference standard** |  |
| Berinson, et al (1) | High risk | Unclear | Unclear | High risk | Low concern | Low concern | High concern | **6** |
| Graff, et al (2) | Low risk | Low risk | Unclear | High risk | Low concern | Unclear | High concern | **8** |
| Holma, et al (3) | Unclear | Unclear | Unclear | Low risk | Low concern | Low concern | Low concern | **11** |
| Sparks, et al (4) | Unclear | Low risk | Low risk | High risk | Low concern | Low concern | Low concern | **11** |
| Cortazzo, et al (5) | Unclear | Unclear | Low risk | Low risk | Low concern | Low concern | Low concern | **12** |
| Peri, et al (6) | High risk | High risk | Low risk | High risk | Low concern | Low concern | Low concern | **8** |
| Sze, et al (7) | High risk | Unclear | Low risk | High risk | Low concern | Low concern | Low concern | **9** |
| Lu, et al (8, 9) | Low risk | Unclear | Unclear | High risk | Low concern | High concern | Low concern | **8** |
| Camelena (10) | Unclear | Unclear | Unclear | Low risk | Low concern | Unclear | Not applicable^a^ | **8** |
| Shah, et al (11) | Low risk | Not applicable^b^ | Low risk^b^ | Low risk | Low concern | Low concern | Not applicable^c^ | **NA** |

For risk of bias assessment: Studies were scored as low risk for a specific domain if answer to all signalling questions of that domain was “yes”; studies were scored as high risk for a specific domain if at least one answer to the signalling questions in that specific domain was no; studies were scored as “unclear” for a specific domain if at least one answer to the signalling questions in that specific domain was “unclear”; if both the answers “no” and “unclear” were used to answer signalling questions in a specific domain, the domain was scored as high risk.

For calculating Quality Score: domains assessed as low risk or low concern were scored as 2, domains assessed as unclear were scored as 1 and domains assessed as high risk or high concern were scored as 0.

^b^Signalling question about blinding not applicable as the study reported data about times to results only; ^c^Signalling question about genotypic confirmation not applicable as the study reported data about times to results only

**Supplementary data 4. Pooled performance characteristics of BCID2 for major clinically relevant determinants of BSI**

| **ENTEROBACTERALES** | | | | | | | | | | | |
| --- | --- | --- | --- | --- | --- | --- | --- | --- | --- | --- | --- |
|  | **TP** | **FP** | **FN** | **TN** | **SE% (95%CI)** | **SP% (95%CI)** | **LR+ (95%CI)** | **LR- (95%CI)** | **DOR (95%CI)** | **AUC (95%CI)** | ***I*^2^ (%)** |
| Berinson et al (1) | 91 | 1 | 1 | 99 | 98.9 (94.1 to 100) | 99.0 (94.6 to 100.0) | 98.91 (14.07 to 695.41) | 0.01 (0.00 to 0.08) | 9009.00 (669.58 to .) | 0.99 (0.98 to 1.00) | - |
| Graff et al (2) | 45 | 0 | 1 | 145 | 97.8 (88.5 to 99.9) | 100.0 (97.5 to 100.0) | . (1 to .) | 0.02 (0.00 to 0.15) | . (874.78 to .) | 0.99 (0.97 to 1.00) | - |
| Holma et al (3) | 53 | 1 | 1 | 68 | 98.1 (90.1 to 100.0) | 98.6 (92.2 to 100.0) | 67.72 (9.67 to 474.12) | 0.02 (0.00 to 0.13) | 3404.00 (265.97 to .) | 0.98 (0.96 to 1.00) | - |
| Sparks et al (4) | 31 | 1 | 1 | 16 | 96.9 (83.8 to 99.9) | 94.1 (71.3 to 99.9) | 16.47 (2.46 to 110.38) | 0.03 (0.00 to 0.23) | 486.00 (35.41 to .) | 0.95 (0.89 to 1.00) | - |
| Cortazzo et al (5) | 54 | 0 | 0 | 36 | 100 (93.4 to 100) | 100 (90.3 to 100) | . (. to .) | 0.00 (. to .) | . (527.41 to .) | 1.00 (1.00 to 1.00) | - |
| Peri et al (6) | 29 | 1 | 1 | 31 | 96.7 (82.8 to 99.9) | 96.9 (83.8 to 99.9) | 30.93 (4.49 to 213.17) | 0.03 (0.01 to 0.24) | 899.00 (65.13 to .) | 0.97 (0.92 to 1.00) | - |
| Sze et al (7) | 33 | 0 | 0 | 28 | 100.0 (89.4 to 100.0) | 100.0 (89.4 to 100.0) | . (. to .) | 0.00 (. to .) | . (250.63 to .) | 1.00 (1.00 to 1.00) | - |
| Lu et al (8, 9) | 269 | 54 | 1 | 750 | 99.6 (98.0 to 100.0) | 93.3 (91.3 to 94.9) | 14.83 (11.46 to 19.19) | 0.00 (0.00 to 0.03) | 3736.11 (645.85 to .) | 0.96 (0.96 to 0.97) | - |
| Camelena (10) | 64 | 0 | 3 | 96 | 95.5 (87.5 to 99.1) | 100.0 (96.2 to 100.0) | . (. to .) | 0.04 (0.01 to 0.14) | . (413.32 to .) | 0.97 (0.91.4 to 99.4) | - |
| **POOLED** | | | | | **98.2 (96.3 to 99.1)** | **98.3 (96.5 to 99.2)** | **57.83 (29.38 to 113.84)** | **0.02 (0.01 to 0.04)** | **3131.33 (1206.27 to 8128.55)** | **0.982 (0.972 to 0.989)** | **0** |
| **S. AUREUS** | | | | | | | | | | | |
|  | **TP** | **FP** | **FN** | **TN** | **SE% (95%CI)** | **SP% (95%CI)** | **LR+ (95%CI)** | **LR- (95%CI)** | **DOR (95%CI)** | **AUC (95%CI)** | ***I*^2^ (%)** |
| Berinson et al (1) | 16 | 0 | 0 | 176 | 100.0 (79.4 to 100.0) | 100.0 (97.9 to 100.0) | . (. to .) | 0.00 (. to .) | . (766.04 to .) | 1.00 (1.00 to 1.00) | - |
| Graff et al (2) | 34 | 0 | 0 | 157 | 100.0 (89.7 to 100.0) | 100.0 (97.7 to 100.0) | . (. to .) | 0.00 (. to .) | . (1448.92 to .) | 100.0 (89.7 to 100.0) | - |
| Holma et al (3) | 11 | 0 | 0 | 112 | 100.0 (71.5 to 100.0) | 100.0 (96.8 to 100.0) | . (. to .) | 0.00 (. to .) | . (336.31 to .) | 1.00 (1.00 to 1.00) | - |
| Sparks et al (4) | 2 | 0 | 0 | 47 | 100.0 (15.8 to 100.0) | 100.0 (92.5 to 100.0) | . (. to .) | 0.00 (. to .) | . (30.05 to .) | 1.00 (1.00 to 1.00) | - |
| Cortazzo et al (5) | 9 | 0 | 0 | 81 | 100.0 (66.4 to 100.0) | 100.0 (95.5 to 100.0) | . (. to .) | 0.00 (. to .) | . (199.50 to .) | 1.00 (1.00 to 1.00) | - |
| Peri et al (6) | 7 | 0 | 0 | 55 | 100.0 (59.0 to 100.0) | 100.0 (93.5 to 100.0) | . (. to .) | 0.00 (. to .) | . (105.88 to .) | 1.00 (1.00 to 1.00) | - |
| Sze et al (7) | 5 | 0 | 0 | 56 | 100.0 (47.8 to 100.0) | 100.0 (93.6 to 100.0) | . (. to .) | 0.00 (. to .) | . (78.18 to .) | 1.00 (1.00 to 1.00) | - |
| Lu et al (8, 9) | 149 | 2 | 0 | 923 | 100.0 (97.6 to 100.0) | 99.8 (99.2 to 100.0) | . (. to .) | 0.01 (0.00 to 0.05) | . (12397.67 to .) | 0.99 (0.98 to 1.00) | - |
| Camelena (10) | 4 | 0 | 1 | 158 | 80.0 (28.4 to 99.4) | 100.0 (97.7 to 100.0) | . (. to .) | 0.20 (0.03 to 1.15) | . (84,41 to .) | 0.90 (0.70 to 1.00) | - |
| **POOLED** | | | | | **96.0 (90.4 to 98.4)** | **99.5 (98.7 to 99.8)** | **204.14 (75.92 to 548.86)** | **0.04 (0.02 to 0.09)** | **5098.42 (1456.31 to 17849.18)** | **0.986 (0.974 to 0.993)** | **0** |
| **STREPTOCOCCUS spp.** | | | | | | | | | | | |
|  | **TP** | **FP** | **FN** | **TN** | **SE% (95%CI)** | **SP% (95%CI)** | **LR+ (95%CI)** | **LR- (95%CI)** | **DOR (95%CI)** | **AUC (95%CI)** | ***I*^2^ (%)** |
| Berinson et al (1) | 27 | 0 | 0 | 165 | 100.0 (87.2 to 100.0) | 100.0 (97.8 to 100.0) | . (. to .) | 0.00 (. to .) | . (1209.64 to .) | 1.00 (1.00 to 1.00) | - |
| Graff et al (2) | 28 | 0 | 0 | 163 | 100.0 (87.7 to 100.0) | 100.0 (97.8 to 100.0) | . (. to .) | 0.00 (. to .) | . (1239.15 to .) | 1.00 (1.00 to 1.00) | - |
| Holma et al (3) | 28 | 1 | 0 | 94 | 100.0 (87.7 to 100.0) | 98.9 (94.3 to 100.0) | 95.00 (13.52 to 667.47) | 0.00 (. to .) | . (351.44 to .) | 0.99 (0.98 to 1.00) | - |
| Sparks et al (4) | 6 | 0 | 0 | 43 | 100.0 (54.1 to 100.0) | 100.0 (91.8 to 100.0) | . (. to .) | 0.00 (. to .) | . (71.28 to .) | 1.00 (1.00 to 1.00) | - |
| Cortazzo et al (5) | 1 | 0 | 0 | 89 | 100.0 (2.5 to 100.0) | 100.0 (95.9 to 100.0) | . (. to .) | 0.00 (. to .) | . (0.00 to .) | 1.00 (. to 1.00) | - |
| Peri et al (6) | 9 | 0 | 0 | 53 | 100.0 (66.4 to 100.0) | 100.0 (93.3 to 100.0) | . (. to .) | 0.00 (. to .) | . (130.39 to .) | 1.00 (1.00 to 1.00) | - |
| Sze et al (7) | 4 | 0 | 0 | 57 | 100.0 (39.8 to 100.0) | 100.0 (93.7 to 100.0) | . (. to .) | 0.00 (. to .) | . (64.77 to .) | 1.00 (1.00 to 1.00) | - |
| Lu et al (8, 9) | 121 | 2 | 2 | 949 | 98.4 (94.2 to 99.8) | 99.8 (99.2 to 100.0) | 467.77 (117.14 to 1867.97) | 0 0.2 (0.00 to 0.06) | 28707.25 (4306.66 to 1.9e+05) | 0.99 (0.98 to 1.00) | - |
| Camelena (10) | 14 | 1 | 1 | 147 | 93.3 (68.1 to 99.8) | 99.3 (96.3 to 100.0) | 138.13 (19.50 to 978.70) | 0.07 (0.01 to 0.45) | 2058.00 (148.74 to .) | 0.96 (0.90 to 1.00) | - |
| **POOLED** | | | | | **96.7 (92.8 to 98.5)** | **99.5 (98.8 to 99.8)** | **188.30 (80.95 to 438.03)** | **0.03 (0.017 to 0.07)** | **5660.11 (1913.79 to 16740.01)** | **0.978 (0.978 to 0.992)** | **0** |
| ***blaCTX-M*** | | | | | | | | | | | |
|  | **TP** | **FP** | **FN** | **TN** | **SE% (95%CI)** | **SP% (95%CI)** | **LR+ (95%CI)** | **LR- (95%CI)** | **DOR (95%CI)** | **AUC (95%CI)** | ***I*^2^ (%)** |
| Berinson et al (1) | 21 | 1 | 0 | 170 | 100.0 (83.9 to 100.0) | 99.4 (96.8 to 100.0) | 171.00 (24.23 to 1206.99) | 0.00 (. to .) | . (476.34 to .) | 1.00 0.99 to 1.00) | - |
| Graff et al (2) | 5 | 0 | 0 | 186 | 100.0 (47.8 to 100.0) | 100.0 (98.0 to 100.0) | . (. to .) | 0.00 (. to .) | . (260.71 to .) | 1.00 (1.00 to 1.00) | - |
| Holma et al (3) | 8 | 0 | 0 | 115 | 100.0 (63.1 to 100.0) | 100.0 (98.0 to 100.0) | . (. to .) | 0.00 (. to .) | . (408.83 to .) | 1.00 (1.00 to 1.00) | - |
| Sparks et al (4) | 2 | 2 | 0 | 45 | 100.0 (15.8 to 100.0) | 95.7 (85.5 to 99.5) | 23.50 (6.06 to 91.20) | 0.00 (. to .) | . (8.10 to .) | 0.98 (0.95 to 1.00) | - |
| Cortazzo et al (5) | 16 | 0 | 0 | 74 | 100.0 (79.4 to 100.0) | 100.0 (95.1 to 100.0) | . (. to .) | 0.00 (. to .) | . (321.83 to .) | 1.00 (1.00 to 1.00) | - |
| Peri et al (6) | 3 | 0 | 0 | 59 | 100.0 (29.2 to 100.0) | 100.0 (93.9 to 100.0) | . (. to .) | 0.00 (. to .) | . (52.07 to .) | 1.00 (1.00 to 1.00) | - |
| Sze et al (7) | 13 | 0 | 0 | 48 | 100.0 (75.3 to 100.0) | 100.0 (92.6 to 100.0) | . (. to .) | 0.00 (. to .) | . (169.80 to .) | 1.00 (1.00 to 1.00) | - |
| Lu et al (8, 9) | 46 | 0 | 0 | 1028 | 100.0 (92.3 to 100.0) | 100.0 (99.6 to 100.0) | . (. to .) | 0.00 (. to .) | . (12836.07 to .) | 1.00 (1.00 to 1.00) | - |
| **POOLED** | | | | | **94.9 (85.7 to 98.3)** | **99.4 (97.8 to 99.8)** | **147.16 (43.79 to 494.55)** | **0.05 (0.02 to 0.1)** | **2870.64 (621.04 to 13268.91)** | **0.982 (0.961 to 0.991)** | **23.9** |
| **CARBAPENEMASES^a^** | | | | | | | | | | | |
|  | **TP** | **FP** | **FN** | **TN** | **SE% (95%CI)** | **SP% (95%CI)** | **LR+ (95%CI)** | **LR- (95%CI)** | **DOR (95%CI)** | **AUC (95%CI)** | ***I*^2^ (%)** |
| Berinson et al (1) | 13 | 0 | 0 | 179 | 100.0 (75.3 to 100.0) | 100.0 (98.0 to 100.0) | . (. to .) | 0.00 (. to .) | . (634.03 to .) | 1.00 (1.00 to 1.00) | - |
| Graff et al (2) | 0 | 0 | 0 | 191 | - | - | - | - | - | - | - |
| Holma et al (3) | 11 | 0 | 0 | 112 | 100.0 (71.5 to 100.0) | 100.0 (96.8 to 100.0) | . (. to .) | 0.00 (. to .) | . (336.31 to .) | 1.00 (1.00 to 1.00) | - |
| Sparks et al (4) | 0 | 0 | 0 | 49 | - | - | - | - | - | - | - |
| Cortazzo et al (5) | 19 | 0 | 0 | 71 | 100.0 (82.4 to 100.0) | 100.0 (94.9 to 100.0) | . (. to .) | 0.00 (. to .) | . (366.40 to .) | 1.00 (1.00 to 1.00) | - |
| Peri et al (6) | 0 | 0 | 0 | 62 | - | - | - | - | - | - | - |
| Sze et al (7) | 7 | 0 | 0 | 54 | 100.0 (59.0 to 100.0) | 100.0 (93.4 to 100.0) | . (. to .) | 0.00 (. to .) | . (103.94 to .) | 1.00 (1.00 to 1.00) | - |
| Lu et al (8, 9) | 9 | 0 | 0 | 1065 | 100.0 (66.4 to 100.0) | 100.0 (99.7 to 100.0) | . (. to .) | 0.00 (. to .) | . (2628.79 to .) | 1.00 (1.00 to 1.00) | - |
| **POOLED** | | | | | **94.9 (83.4 to 98.6)** | **99.7 (98.9 to 99.9)** | **364.68 (85.82 to 1549.70)** | **0.05 (0.02 to 0.14)** | **7158.33 (1213.56 to 42224.26)** | **0.988 (0.972 to 0.995)** | **0** |
| ***mecA/C & MREJ*** | | | | | | | | | | | |
|  | **TP** | **FP** | **FN** | **TN** | **SE% (95%CI)** | **SP% (95%CI)** | **LR+ (95%CI)** | **LR- (95%CI)** | **DOR (95%CI)** | **AUC (95%CI)** | ***I*^2^ (%)** |
| Berinson et al (1) | 0 | 0 | 0 | 192 | - | - | - | - | - | - | - |
| Graff et al (2) | 6 | 0 | 0 | 185 | 100.0 (54.1 to 100.0) | 100.0 (98.0 to 100.0) | . (. to .) | 0.00 (. to .) | . (308.15 to .) | 1.00 (1.00 to 1.00) | - |
| Holma et al (3) | 9 | 0 | 0 | 114 | 100.0 (66.4 to 100.0) | 100.0 (96.8 to 100.0) | . (. to .) | 0.00 (. to .) | . (280.96 to .) | 1.00 (1.00 to 1.00) | - |
| Sparks et al (4) | 1 | 0 | 0 | 48 | 100.0 (2.5 to 100.0) | 100.0 (92.6 to 100.0) | . (. to .) | 0.00 (. to .) | . (0.00 to .) | 1.00 (. to 1.00) | - |
| Cortazzo et al (5) | 5 | 0 | 0 | 85 | 100.0 (47.8 to 100.0) | 100.0 (95.8 to 100.0) | . (. to .) | 0.00 (. to .) | . (118.90 to .) | 1.00 (1.00 to 1.00) | - |
| Peri et al (6) | 0 | 0 | 0 | 62 | - | - | - | - | - | - | - |
| Sze et al (7) | 3 | 0 | 0 | 58 | 100.0 (29.2 to 100.0) | 100.0 (93.8 to 100.0) | . (. to .) | 0.00 (. to .) | . (51.18 to .) | 1.00 (1.00 to 1.00) | - |
| Lu et al (8, 9) | 52 | 2 | 0 | 1020 | 100.0 (93.2 to 100.0) | 98.1 (93.2 to 100.0) | 52.0 (13.18 to 205.15) | 0.00 (. to .) | . (474.83 to .) | 0.99 (0.98 to 1.00) | - |
| **POOLED** | | | | | **93.9 (83.0 to 98.0)** | **99.6 (98.6 to 99.9)** | **236.42 (66.5 to 840.52)** | **0.06 (0.03 to 0.15)** | **3845.75 (809.10 to 18279.33)** | **0.984 (0.966 to 0.993)** | **0** |

TP = True Positives; FP = False Positives; FN = False Negatives; TN = True Negatives; SE = Sensitivity; SP = Specificity; LR = Likelihood Ratio; DOR = Diagnostic Odds Ratio; AUC = Area Under the Curve

^a^Including *blaKPC, blaIMP, blaOXA-48, blaNDM, blaVIM*

**Supplementary data 5. Doi plots assessing publication bias: A) *Enterobacterales* B) *S. aureus* C) *Streptococcus* spp. D) *bla*_CTX-M_ E) carbapenemases F) *mecA/C & MREJ***


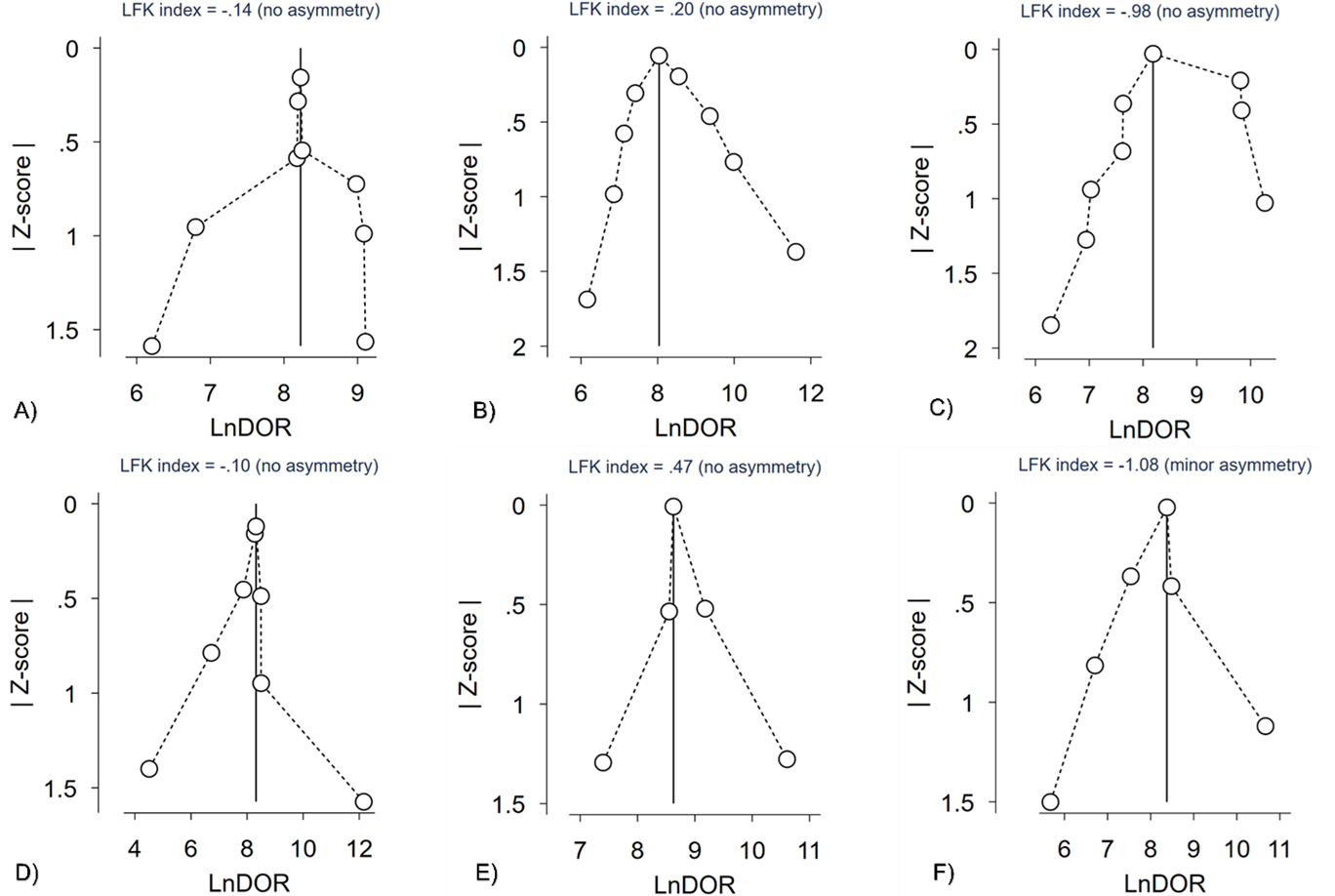


LnDOR = natural logarithm of the diagnostic odds ratio

|  | **SE (95%CI)** | **SP (95%CI)** | **LR+ (95%CI)** | **LR- (95%CI)** | **DOR (95%CI)** | **AUC (95%CI)** | ***I*^2^ (%)** |
| --- | --- | --- | --- | --- | --- | --- | --- |
| ***Enterobacterales*** | 98.2 (96.1 to 99.1) | 98.3 (96.3 to 99.2) | 56.1 (27.86 to 112.961) | 0.02 (0.01 to 0.04) | 2993.52 (1115.94 to 8030.15) | 0.982 (0.971 to 0.989) | 0 |
| ***S. aureus*** | 99.5 (89.0 to 99.2) | 99.5 (98.6 to 99.8) | 194.62 (70.19 to 539.66) | 0.05 (0.02 to 0.1) | 4260.65 (1179.63 to 15388.82) | 0.985 (0.972 to 0.992) | 0 |
| ***Streptococcus spp.*** | 96.0 (91.2 to 98.2) | 99.4 (98.6 to 99.8) | 168.88 (70.03 to 407.27) | 0.04 (0.02 to 0.08) | 4207.96 (1369.11 to 12933.21) | 0.985 (0.974 to 0.991) | 0 |
| ***blaCTX-M*** | 94.7 (84.8 to 98.3) | 99.3 (97.6 to 99.8) | 136.40 (39.52 to 470.77) | 0.05 (0.02 to 0.14) | 2534.28 (530.30 to 12111.27) | 0.981 (0.958 to 0.991) | 23.9 |
| **Carbapenemases** | 94.6 (82.1 to 98.5) | 99.7 (98.8 to 99.9) | 353.41 (79.75 to 1566.16) | 0.05 (0.02 to 0.15) | 6551.25 (1059.48 to 40509.57) | 0.988 (0.97 to 0.995) | 0 |
| ***mecA/C & MREJ*** | 92.7 (80.1 to 97.6) | 99.6 (98.5 to 99.9) | 222.81 (50.52 to 820.37) | 0.07 (0.03 to 0.18) | 3046.45 (622.68 to 14904.67) | 0.982 (0.961 to 0.992) | 0 |

**Supplementary data 6. Pooled performance characteristics of BCID2 combined for main BSI targets according to the Quality Effect Model**

SE = Sensitivity; SP = Specificity; LR = Likelihood Ratio; DOR = Diagnostic Odds Ratio; AUC = Area Under the Curve

References

1. Berinson B, Both A, Berneking L, Christner M, Lutgehetmann M, Aepfelbacher M, Rohde H. 2021. Usefulness of BioFire FilmArray BCID2 for Blood Culture Processing in Clinical Practice. J Clin Microbiol 59:e0054321.

2. Graff KE, Palmer C, Anarestani T, Velasquez D, Hamilton S, Pretty K, Parker S, Dominguez SR. 2021. Clinical Impact of the Expanded BioFire Blood Culture Identification 2 Panel in a U.S. Children's Hospital. Microbiol Spectr 9:e0042921.

3. Holma T, Torvikoski J, Friberg N, Nevalainen A, Tarkka E, Antikainen J, Martelin JJ. 2021. Rapid molecular detection of pathogenic microorganisms and antimicrobial resistance markers in blood cultures: evaluation and utility of the next-generation FilmArray Blood Culture Identification 2 panel. Eur J Clin Microbiol Infect Dis doi:10.1007/s10096-021-04314-2.

4. Sparks R, Balgahom R, Janto C, Polkinghorne A, Branley J. 2021. Evaluation of the BioFire Blood Culture Identification 2 panel and impact on patient management and antimicrobial stewardship. Pathology doi:10.1016/j.pathol.2021.02.016.

5. Cortazzo V, D'Inzeo T, Giordano L, Menchinelli G, Liotti FM, Fiori B, De Maio F, Luzzaro F, Sanguinetti M, Posteraro B, Spanu T. 2021. Comparing BioFire FilmArray BCID2 and BCID Panels for Direct Detection of Bacterial Pathogens and Antimicrobial Resistance Genes from Positive Blood Cultures. J Clin Microbiol 59.

6. Peri AM, Bauer MJ, Bergh H, Butkiewicz D, Paterson DL, Harris PN. 2022. Performance of the BioFire Blood Culture Identification 2 Panel for the Diagnosis of Bloodstream Infections on Blood Cultures from the Intensive Care Unit and Emergency Department. SSRN Electronic Journal doi:10.2139/ssrn.4003859.

7. Sze DTT, Lau CCY, Chan TM, Ma ESK, Tang BSF. 2021. Comparison of novel rapid diagnostic of blood culture identification and antimicrobial susceptibility testing by Accelerate Pheno system and BioFire FilmArray Blood Culture Identification and BioFire FilmArray Blood Culture Identification 2 panels. BMC Microbiol 21:350.

8. Lu Y, Hatch J, Holmberg K, Hurlock A, Drobysheva D, Spaulding U, Vourli S, Pournaras S, Everhart K, Leber A, Barr B, Daly J, Henry T, Johnson A, Balada-Llasat J, Rhoads D, Jacobs M, McKinley K, Harrington A, Zhang F, Berry G, Jeong M, She R, Fantini M, Dirani G, Zannoli S, Sambri V, Bourzac K. 2019. P651. Multi-center Evaluation of the BioFire® FilmArray® Blood Culture Identification 2 Panel for the Detection of Microorganisms and Resistance Markers in Positive Blood Cultures, abstr IDWeek, Washington DC, U.S.,

9. bioMérieux. 2021. BioFire® Blood Culture Identification 2 (BCID2) Panel, Instructions for use and manual. <https://www.biofiredx.com/e-labeling/ITI0048>. Accessed April, 19th 2021.

10. Camelena F. 2021. Performances et impact thérapeutique du BioFire Blood Culture Identification 2 (BCID2) Panel au cours du sepsis, abstr 23es Journées Nationales d'Infectiologie, Monpellier, France, August 30th, September 1st, 2021.

11. Shah S, Davar N, Thakkar P, Sawant C, Jadhav L. 2022. Clinical utility of the FilmArray blood culture identification 2 panel in identification of microorganisms and resistance markers from positive blood culture bottles. Indian J Microbiol Res 9:28-33.
